# Long-term evolution of the epithelial cell secretome in preclinical 3D models of the human bronchial epithelium

**DOI:** 10.1038/s41598-021-86037-0

**Published:** 2021-03-23

**Authors:** Daniel Sanchez-Guzman, Sonja Boland, Oliver Brookes, Claire Mc Cord, René Lai Kuen, Valentina Sirri, Armelle Baeza Squiban, Stéphanie Devineau

**Affiliations:** 1 Université de Paris, BFA, UMR 8251, CNRS, 75013 Paris, France; 2 Cellular and Molecular Imaging Facility, US25 Inserm-3612 CNRS, Faculté de Pharmacie de Paris, Université de Paris, Paris, France

**Keywords:** Cell biology, Proteomics, Respiratory system models

## Abstract

The human bronchial epithelium is the first line of defense against atmospheric particles, pollutants, and respiratory pathogens such as the novel SARS-CoV-2. The epithelial cells form a tight barrier and secrete proteins that are major components of the mucosal immune response. Functional in vitro models of the human lung are essential for screening the epithelial response and assessing the toxicity and barrier crossing of drugs, inhaled particles, and pollutants. However, there is a lack of models to investigate the effect of chronic exposure without resorting to animal testing. Here, we developed a 3D model of the human bronchial epithelium using Calu-3 cell line and demonstrated its viability and functionality for 21 days without subculturing. We investigated the effect of reduced Fetal Bovine Serum supplementation in the basal medium and defined the minimal supplementation needed to maintain a functional epithelium, so that the amount of exogenous serum proteins could be reduced during drug testing. The long-term evolution of the epithelial cell secretome was fully characterized by quantitative mass spectrometry in two preclinical models using Calu-3 or primary NHBE cells. 408 common secreted proteins were identified while significant differences in protein abundance were observed with time, suggesting that 7–10 days are necessary to establish a mature secretome in the Calu-3 model. The associated Reactome pathways highlight the role of the secreted proteins in the immune response of the bronchial epithelium. We suggest this preclinical 3D model can be used to evaluate the long-term toxicity of drugs or particles on the human bronchial epithelium, and subsequently to investigate their effect on the epithelial cell secretions.

## Introduction

With a daily inhaled volume of 10 m^3^ per individual, the respiratory tract is the main route of exposure to atmospheric particles, pollutants, and respiratory pathogens. The epithelia that line the respiratory tract are covered by lung lining fluid, consisting of mucus in the upper airways and surfactant in the alveoli, which exhibits mechanical, antimicrobial, and antioxidant functions to protect the epithelium^1^. The inhalation of exogenous compounds (drugs, atmospheric particles, pollutants) or pathogens (bacteria, viruses) can induce pro-inflammatory epithelial response, release of danger signals to the immune system, cell migration, and cell death^2,3^. The agent can also translocate through the intact or damaged epithelial barrier causing potential systemic effects^4,5^.

The toxicological assessment of drugs and particles following exposure by inhalation has long relied on in vivo studies^6,7^. However, the limited number of compounds that can be tested, the interspecies variability, the associated cost and ethical concerns have supported the development of alternative in vitro models of the human upper and lower airways^8–10^. Functional bronchial epithelium models were developed to investigate the toxicity and translocation of various agents^11,12^. These models have greatly promoted our understanding of the epithelial response and supported the identification of the adverse outcome pathways of toxic compounds.

The available models of the human bronchial epithelium using cell lines can be used to study acute toxicity following short-term exposure, usually 24 or 48 h^13,14^. However there is a lack of functional models to investigate the long-term toxicity of drugs or pollutants following chronic exposure or repeated exposure at low doses. The difficulties associated with long-term cell cultures (without subculturing) are decreased cell viability, divergence and changes in cellular features, and stratification due to continuous proliferation. The development of in vitro lung models to investigate the chronic toxicity of exogenous agents by inhalation is therefore a key step in the field of predictive toxicology.

Primary normal human bronchial epithelial (NHBE) cells collected from healthy donors are commercially available. NHBE cells can be cultured on porous membranes (inserts) at the air liquid interface (ALI), which leads to their differentiation into a mucociliary epithelium after 3–4 weeks. Composed of epithelial cells, including ciliated and goblet cells, these cultures can be maintained for several weeks at ALI with minor modifications^15^. They represent one of the best options to evaluate the long-term effect of a compound on the bronchial epithelium. However, high cost, low rate of cell expansion before differentiation, time required to establish a stable differentiated epithelium, and donor variability, limit their use for high throughput toxicological screening^16,17^.

The human Calu-3 lung adenocarcinoma cell line (ATCC HTB-55) is an interesting alternative to primary cells for the development of robust and low-cost models of the human bronchial epithelium for toxicological assessment. The Calu-3 cells differentiate into a functional tight epithelium when cultured on inserts at ALI. Even though the resulting epithelium is limited to one cell type, it exhibits features specific to the human bronchial epithelium such as a tight epithelial barrier, mucus secretion, receptor expression, and cytokine production among others^9,18–23^. Expert panels and pre-validation assays have put forward the Calu-3 model of the bronchial epithelium for drug screening^24–26^. Readers may refer to reviews for more details about this cell line^25,27–29^. Other lung cell lines could be used as in vitro models, such as the epithelial BEAS-2B, NCI-H292, and 16HBE cell lines^30–32^. Contrary to BEAS-2B, NCI-H292, and 16HBE, the Calu-3 cell line can simultaneously form a tight epithelium at ALI and secrete mucus^33^. Hence, we selected the Calu-3 cell line for this study.

The mucus secreted by airway epithelial cells plays a key role in the protection against and clearance of particles and pathogens^34^. Secreted proteins in the mucus are involved in the cell-to-cell communication and the mucosal immune response^35^. Changes in cell secretions are associated with pulmonary diseases including COPD, asthma, and lung cancers^36,37^. However, little is known about the evolution of the epithelial cell secretion with time, especially for long periods (2–4 weeks) studied in the context of chronic toxicity studies. In addition, the drug or the inhaled particles may alter the epithelial cell secretion profiles, which is an important element of the epithelium response^38^.

Here, we developed a 3D model of the human bronchial epithelium using Calu-3 cells and investigated its viability, morphology, and functionality for 21 days following differentiation at ALI. Barrier function of the epithelium, protein secretion, and the composition of the apical secretome were monitored over time in the Calu-3 model of the bronchial epithelium. Reduced FBS (fetal bovine serum) supplementation was tested in the basal medium in long-term cell cultures. Minimizing the intake of exogenous proteins is important for the long-term toxicological study of both drugs and particles. FBS is composed of more than 1800 proteins and 4000 metabolites with high batch-to-batch variability^39^. Some drugs can form complexes with serum protein, which changes their bioavailability. Nanoparticles are prone to adsorb proteins on their surface, forming a so-called protein corona that drives cell-nanoparticle interactions^40,41^, an effect that is amplified when cells and serum from different species are used^42^. A minimal FBS supplementation of 4% was defined for long-term cell cultures in our Calu-3 model.

The epithelial cell secretions of models based on Calu-3 and on primary NHBE cells from three healthy donors were characterized from 4 to 18 days at ALI by quantitative mass spectrometry. We observed that the same proteins were secreted in both models, albeit with differences in abundance with time and between models. The time evolution of the secretome of the Calu-3 cells shows that a stable and mature secretome was established after 7–10 days at ALI. The Reactome pathway analysis of the Calu-3 and NHBE secretome highlighted the role of the secreted proteins in the mucosal immune response in vitro. We suggest this pre-clinical model can be used to evaluate the chronic toxicity of drugs, particles or pollutants following single or repeated exposure by inhalation, and to investigate their effect on the epithelial cell secretions.

## Results

### Long-term cultures of Calu-3 cells at the air–liquid interface with minimal FBS supplementation

**Figure 1:**
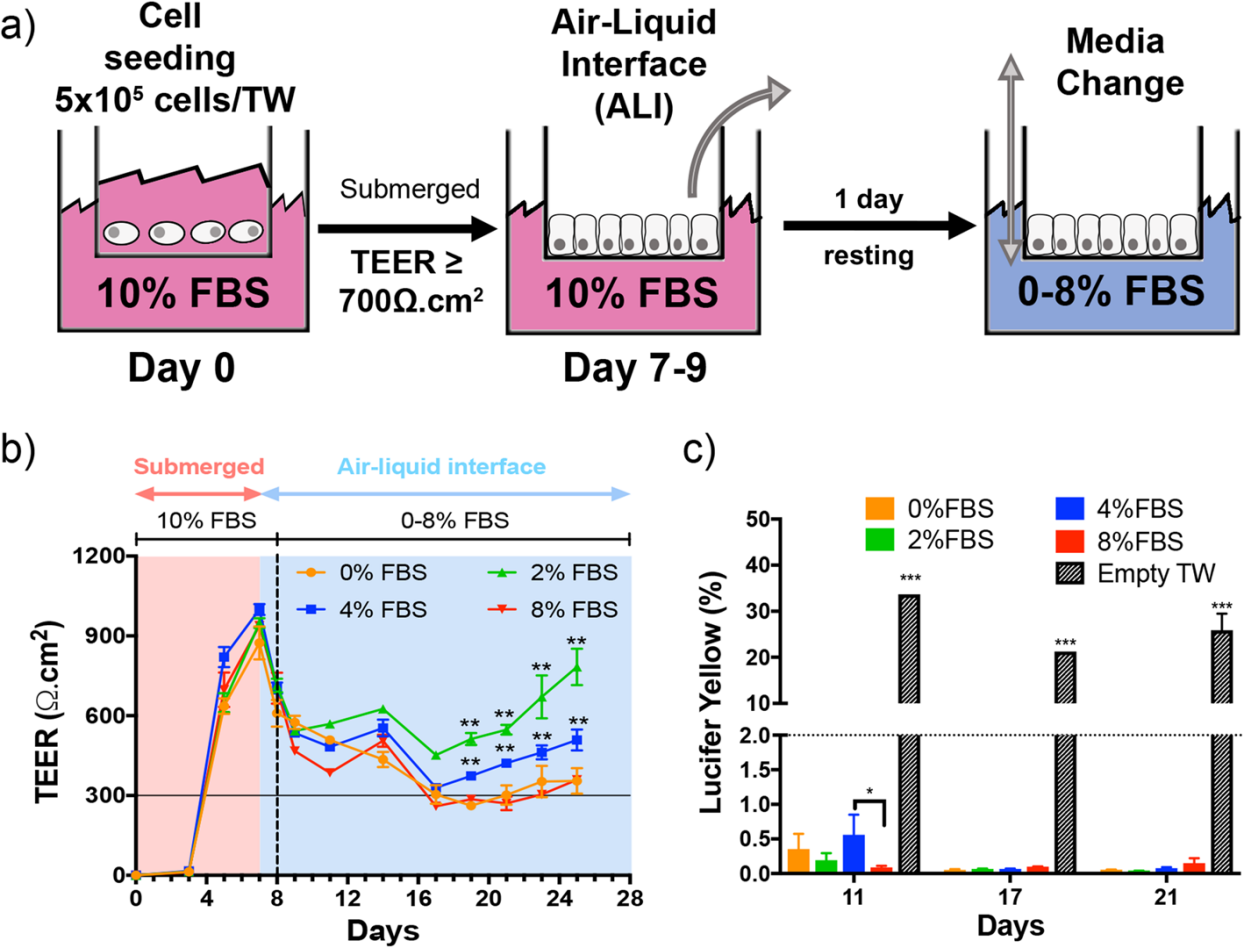
Development of a 3D model of the human bronchial epithelial using Calu-3 cells for long-term toxicity studies. (**a**) Schematic of the experimental protocol to establish long-term cultures of Calu-3 cells. Cells were grown on Transwell inserts with a 3-µm pore diameter. Cell differentiation was induced by changing culture conditions from submerged to ALI. The barrier integrity of the epithelium was assessed by the measurement of the trans-epithelial electrical resistance (TEER) and the paracellular permeability (Lucifer Yellow (LY) permeability assay). (**b**) Evolution of the TEER for 21 days in a representative experiment (n = 3). A TEER > 300 Ω cm^2^ (horizontal line) is associated with a tight epithelium (n = 3). (**c**) Measurement of the paracellular permeability with the LY assay at 11, 17, and 21 days. An empty Transwell insert was used as a negative control. A LY permeability < 2% (dotted line) is associated with a tight epithelium (n = 3). Significant differences between conditions (FBS %) *P < 0.01. **P < 0.05, ***P < 0.001.

First, we developed a protocol to establish a long-term culture of Calu-3 cells at the air–liquid interface (Fig. 1a). Calu-3 cells were grown on Transwell inserts with a 3 µm pore diameter with MEM supplemented with 10% FBS in both the apical and basal compartments (submerged conditions). We did not observe any cell migration through the pores, nor cell growth on the basal side of the insert membrane. The cultures were maintained in submerged conditions for approximately 7 days until a TEER ≥ 700 Ω cm^2^ was obtained, corresponding to a confluent culture. Calu-3 differentiation was initiated by changing culture conditions from submerged to ALI by removing the apical medium, without addition of any inducer. One day after ALI, the basal medium was replaced with MEM containing 0–8% FBS corresponding to our experimental conditions. We used these conditions to determine the effect of FBS concentration on the viability and functionality of the human bronchial epithelium in our 3D model for a 4-week period. The objective was to define the minimal FBS supplementation needed to maintain a functional epithelium over a 4-week period so that the amount of exogenous serum proteins could be reduced during drug testing.

The formation of the epithelial barrier was monitored by measuring the trans-epithelial electrical resistance (TEER) before ALI and for a 4-week period (Fig. 1b). The TEER increases with the formation of tight junctions, which leads to a reduction in paracellular ion/electron transport. A TEER > 300 Ω cm^2^ is associated with a tight epithelium at ALI^43^. We observed an increase of the TEER with a maximum value of 940 ± 69 Ω cm^2^ at day 7 in submerged conditions. At day 8 (day 1 after ALI), the TEER decreased to 682 ± 76 Ω cm^2^. A TEER > 300 Ω cm^2^ was maintained for at least 18 days after establishing ALI (corresponding to a total number of 25 days in culture) (Figs. 1b, S1). Interestingly, a TEER equal or higher to this minimal value was measured even without any FBS in the basal medium. The highest TEER value was observed with 2% FBS. Similar values were measured with 0, 4, and 8% FBS. These results show that a decrease of FBS percentage in the basal medium does not alter the barrier integrity of the bronchial epithelium in long-term cultures. We monitored the TEER of two additional Calu-3 models prepared at different cell passages to check for the variability of the TEER measurement in Calu-3 cultures (Fig. S1). The three biological replicates shown in Fig. S1 correspond to Calu-3 cells thawed and cultured independently on different days. Similar results were obtained with a TEER > 300 Ω cm^2^ in all the experiments up to 21 days after ALI.

The paracellular permeability of the epithelium was measured using the Lucifer Yellow (LY) permeability assay to confirm the TEER results (Fig. 1c). A LY permeability < 2% was measured in all conditions, confirming the integrity of the bronchial epithelium for 21 days with reduced FBS supplementation in the basal medium. We compared the TEER measurement and LY permeability of 236 different samples with tight or damaged epithelial barrier (Fig. S2). This broad analysis confirmed the minimal TEER value of 300 Ω cm^2^ defined for a tight epithelium in our experimental conditions. Therefore, the variations of the TEER above this limit (e.g. between 300 and 800 Ω cm^2^) do not reflect alteration in the barrier function of the epithelium. Higher values may be due to differences in the epithelium thickness or in ion secretion^28^. The EdU (5-ethynyl-2′-deoxyuridine) cell proliferation assay confirmed that Calu-3 cells can proliferate with reduced FBS supplementation in the basal medium (Fig. S3).

**Figure 2:**
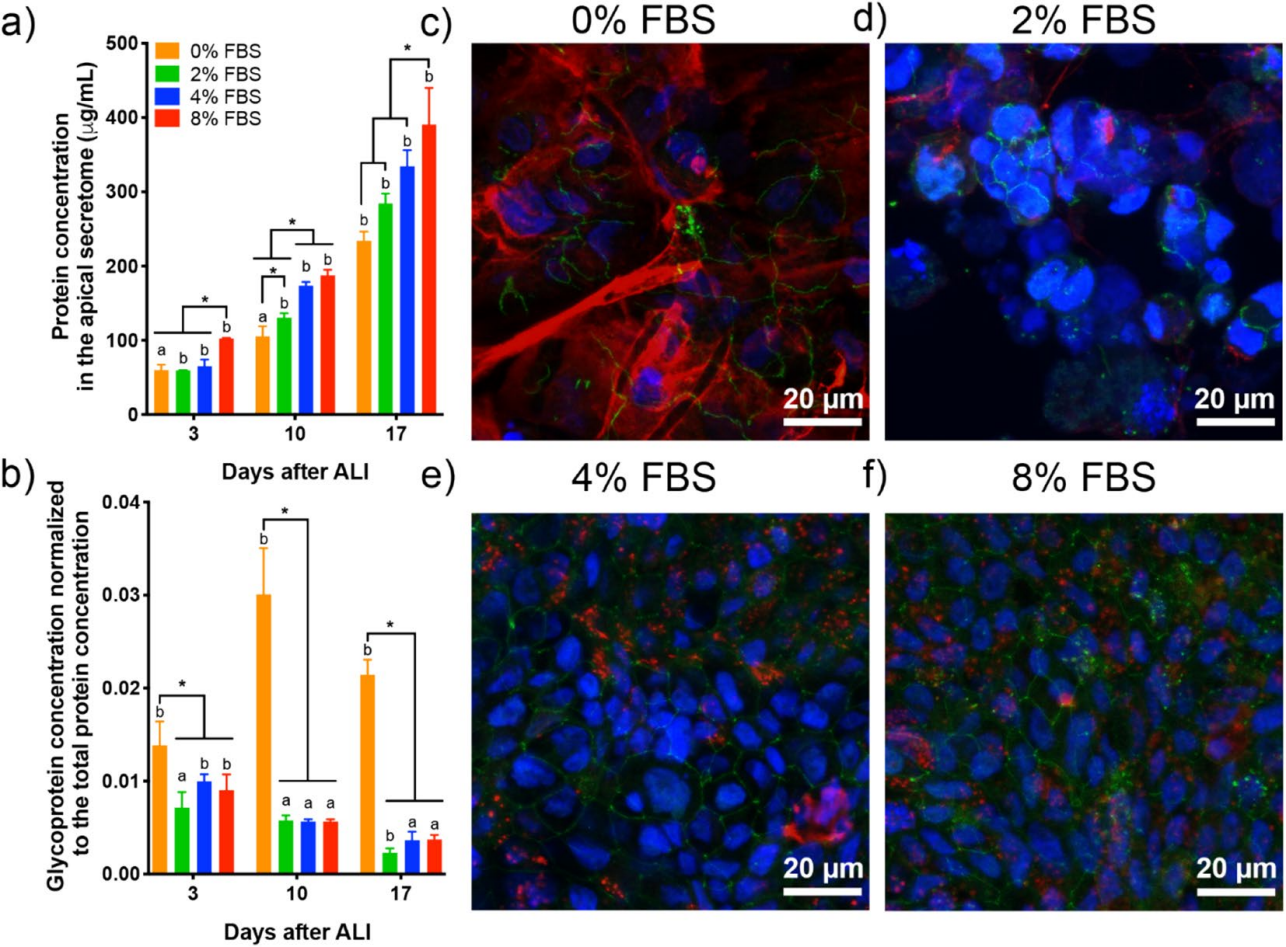
Effect of lower FBS supplementation on the apical secretion by Calu-3 cells in long-term cultures. (**a**) Total protein and (**b**) glycoprotein concentration in the apical secretome of Calu-3 cells supplemented with 0, 2, 4, 8% FBS at day 3, 10 and 17 after ALI. The glycoprotein concentration was normalized to the total protein concentration. (**c**–**f**) Immunolabelling of ZO-1 (green) and MUC5AC (red) in DAPI-stained Calu-3 cells at day 17 after ALI. The cell cultures were supplemented with 0 (**c**), 2 (**d**), 4 (**e**), and 8% FBS. Each image is the z-projection of 10 slides. (n = 3) *P < 0.01 ^a,b^statistically different between days.

Then, we studied the effect of reduced FBS supplementation on protein secretion by epithelial cells for 17 days at ALI. The total protein and glycoprotein concentrations of the apical secretome of the Calu-3 cells were measured at day 3, 10, and 17 after ALI (Fig. 2a,b). The apical secretome was collected every 2 days in this experiment to avoid bias due to protein accumulation. We observed a significant increase in protein secretion both with time and with FBS percentage (Fig. 2a). This result may reflect an increasing metabolic activity of Calu-3 cells at ALI with time, and a reduced metabolic activity when cells have a lower FBS supply. However, a different trend was observed when looking at glycoproteins specifically, among which mucins would be the most abundant. The glycoprotein concentration was normalized to the total protein concentration at each day. A steady normalized glycoprotein concentration was measured over time for the 2, 4, 8% FBS conditions, while a significant increase was observed without supplementation (Fig. 2b). This result indicates that glycoprotein secretion by the epithelial cells is stable once the epithelial barrier is well established for cells cultured with reduced FBS supplementation, while a lack of FBS induces hypersecretion of glycoproteins by Calu-3 cells at ALI.

To determine whether glycoprotein secretion included mucin secretion in the apical secretome, immunolabelling and confocal microscopy imaging of the gel-forming mucin MUC5AC and the tight-junction associated protein zonula occludens-1 (ZO-1) was performed on Calu-3 cells 17 days after ALI (Fig. 2c–f). MUC5AC staining appears as small dots distributed in the apical region of the cell for the 0, 2, 4, 8% FBS conditions, confirming mucus secretion in long-term Calu-3 cell cultures at ALI. Mucus secretion is usually partially or totally lost during the washes in the cell fixation protocol. Similar MUC5AC staining was observed with 4 and 8% FBS supplementation. ZO-1 staining confirmed the presence of tight junction in long-term Calu-3 cultures, making them a good model of the human bronchial epithelium for chronic toxicity studies with reduced FBS supplementation.

A distinct MUC5AC staining was observed in the case of Calu-3 cells cultured without FBS. Large patches of MUC5AC were visible on the epithelium surface, forming thick mucus filaments. The hypersecretion of mucin was confirmed by qRT-PCR with the up-regulation of *muc5ac* in Calu-3 cells deprived of FBS compared to cells cultured with 10% FBS in the basal medium (Fig. S4). Up-regulation of serum albumin (*alb*) and the tight junction associated protein, zonula occludens-1 (*zo1*), was also observed. Surprisingly, significant down-regulation of *muc5b* was seen in Calu-3 cells supplemented with 2% FBS, while no change occurred at 0% suggesting that mucus hypersecretion was mediated by MUC5AC in conditions without FBS. This feature is reminiscent of mucus hypersecretion described in respiratory diseases, including asthma, COPD, and lung cancers^44–46^. In our model, the hypersecretion of MUC5AC by Calu-3 cells, derived from a lung adenocarcinoma, was induced by deprivation of any FBS supplementation only, while no visible over-production of MUC5AC was observed in long-term cell cultures with reduced FBS supplementation (Fig. 2).

We observed that Calu-3 cells could be maintained for 18 days at ALI with reduced FBS supplementation. With 4% FBS supplementation in the basal medium, Calu-3 cells exhibit good functionality and viability, as evidenced by the TEER measurement, the LY permeability assay, the analysis of the protein secretion, and MUC5AC immunolabelling. Transmission electron microscopy images of the bronchial epithelium formed by Calu-3 cells cultured with a reduced FBS supplementation of 4% are shown 8 days after ALI in Fig. S5. Calu-3 cells formed an epithelium monolayer with visible tight junctions and a high number of secretory vesicles in some cells^33^. The release of mucus vesicles (or granules) at the apical side of the epithelium was also observed (Fig. S5e). Based on these observations, we chose a minimal FBS supplementation of 4% to characterize the epithelial cell secretions in a 3D model of the human bronchial epithelium in the second part of this study.

### Quantitative proteomic analysis of the secretome in Calu-3 and NHBE models

We characterized the secretome of Calu-3 cells in long-term cell cultures by label-free quantitative mass spectrometry. The apical secretome was collected in Hanks’ balanced salt solution (HBSS) at day 4, 11, and 18 after ALI for analysis. We performed the same experiment with NHBE cells to compare the composition of the epithelial cell secretions in Calu-3 and NHBE models of the human bronchial epithelium. All the experiments were performed in biological and technical triplicates. In the case of primary cells, NHBE cells from 3 healthy donors were used and the apical secretome collected in HBSS at day 4, 12, and 18 after ALI. The NHBE cells were cultured on inserts with a 0.4 µm pore diameter following the provider’s instructions. The commercial cell culture medium used for NHBE cells is serum free (details on growth factors or other additives were not disclosed by the provider).

**Figure 3:**
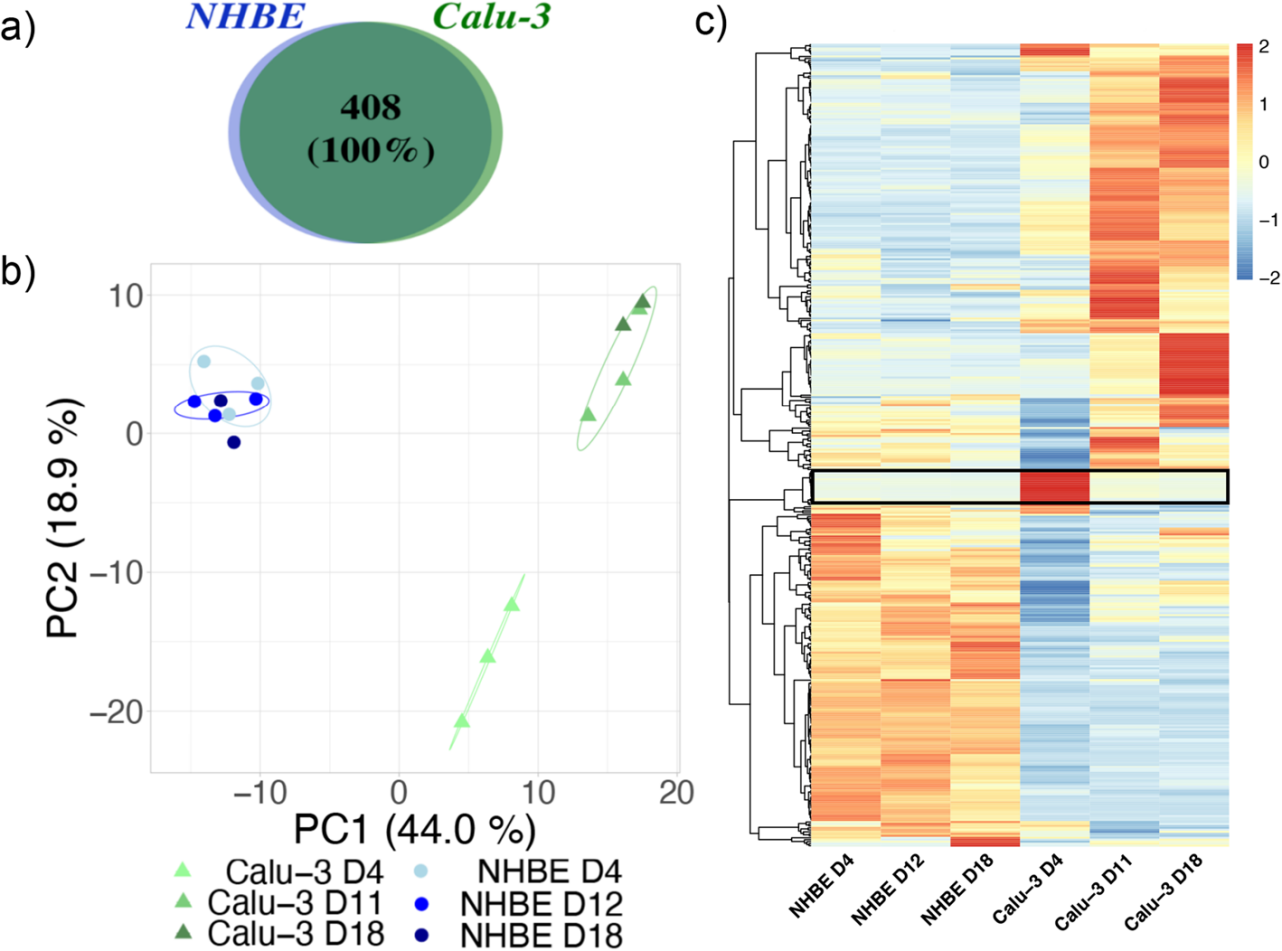
Proteomic analysis of the apical secretome of the human bronchial epithelium in Calu-3 and NHBE models. (**a**) Qualitative analysis of the extracellular proteins of Calu-3 and NHBE cells at day 4, day 11 (Calu-3) or 12 (NHBE), and day 18 after ALI. The total number of proteins and the percentage of common proteins are shown in a Venn diagram. (**b**) Comparison of the abundance of extracellular proteins by Principal Component Analysis (PCA). The analysis was performed at day 4, day 11 (Calu-3) or 12 (NHBE), and day 18. The different donors (NHBE cells) and biological replicates (Calu-3 cells) are represented for each condition and circled on the graph. The percentage associated with each principal component is indicated in the axis legend. (**c**) Heat map of extracellular proteins identified in the apical secretome of Calu-3 cells and NHBE cells at day 4, 11–12, and 18 after ALI. A protein set showing a large difference at day 4 in the secretome of Calu-3 cells is highlighted by a black rectangle.

A total number of 1685 proteins were identified in the apical secretome of Calu-3 and NHBE cells at all time points. The full protein list is presented in the Supplementary dataset 1. These proteins include several intracellular proteins that may originate from dead cells and cell debris, which are collected with the secretome during washes. Extracellular proteins were selected using the Proteome Discover database. We identified 408 extracellular proteins in the apical secretome of Calu-3 and NHBE cells (Fig. 3). Each protein was identified in at least two biological replicates, showing that little difference exists between cell batches (in the case of Calu-3 cell lines) or donors (in the case of NHBE primary cells). Interestingly, the same proteins were identified in the secretome of Calu-3 and NHBE cells, indicating a very high similarity in the epithelial cell secretome in the Calu-3 and NHBE model over time (Fig. 3a).

The abundance of each protein was analyzed so that we could determine whether one protein was more or less secreted in one condition, either between the Calu-3 and the NHBE model, or as a function of time. To get an overview of the proteomic pattern for each condition, a principal component analysis (PCA) of the quantitative proteomic data was performed. The plot of the two main eigenvectors PC1 and PC2 is shown in Fig. 3b. This analysis shows that the secretomes of NHBE cells form a single cluster that differs from the secretome of Calu-3 cells at all time points. No difference in the secretome of NHBE cells with time or between donors was observed in this analysis, suggesting a high stability of the NHBE culture over time and little difference in the secretions of the epithelial cells between donors. Note that both male and female healthy donors of different ages were considered in this study. On the contrary, Calu-3 secretomes form two separate clusters at day 4, and at day 11 and 18 after ALI respectively. This analysis suggests that the protein abundance in the apical secretome of Calu-3 cells may evolve during the first days at ALI until it stabilizes and forms a mature secretome.

The relative abundance of the 408 extracellular proteins identified in the secretome of Calu-3 and NHBE cells is shown in a heat map in Fig. 3c. This analysis confirms the high level of similarity of the apical secretome of NHBE cells as a function of time. On the contrary, larger variations in protein abundances are observed at day 4 compared to day 11 and 18 after ALI in the case of Calu-3 cells. The detailed analysis shows that these differences are usually associated with an increase of the protein concentration with time between day 4 and day 11 after ALI. However, a cluster of proteins showed a different trend, with a higher abundance in the secretome of Calu-3 cells at day 4 compared to day 11 and 18 after ALI (Fig. 3c).

Differences in protein abundances were clearly observed between the secretome of NHBE and Calu-3 cells at each time point. This result shows that, despite a high degree of similarity in terms of protein composition, the epithelial cell secretions differ in their protein abundance and temporal evolution in the Calu-3 and NHBE models.

**Figure 4:**
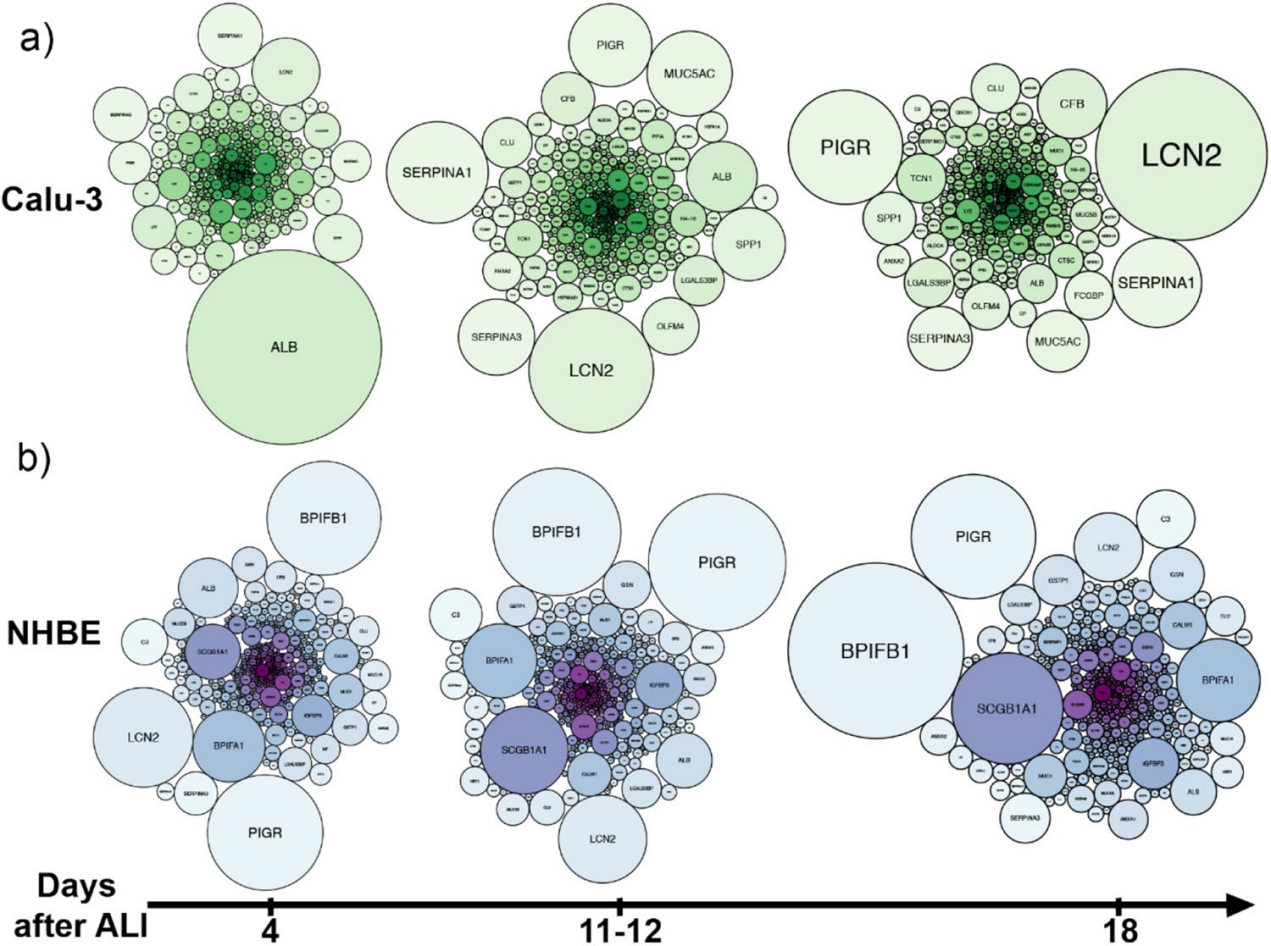
Time evolution of the secretome of Calu-3 and NHBE cells. Schematic representing the proteins secreted by Calu-3 (**a**) and NHBE (**b**) cells as a function of their abundance at day 4, 11–12, and 18 after ALI. A larger sphere denotes a higher protein abundance. Proteins are designated by their corresponding gene. Human serum albumin (*alb*), neutrophil gelatinase-associated lipocalin (*lcn2*), polymeric immunoglobulin receptor (*pigr*), alpha-1-antitrypsin (*serpina1*), BPI fold containing family B member 1 (*bpfib1*). The full protein list is detailed in Table S1.

To get a better view of these differences, the secreted proteins of each model were represented as a function of time using a sphere diagram (Fig. 4). The most abundant protein in the secretome of Calu-3 cells at day 4 was serum albumin (ALB), which abundance then decreases at day 11 and 18 after ALI. The potential contamination of the apical secretome by bovine serum albumin from the basal medium was checked using two methods: first, the peptide sequence analysis showed higher sequence homology with *Homo sapiens* versus *Bos Taurus* serum albumin using the Swissprot database; secondly, up-regulation of HSA expression in Calu-3 cells was evidenced by qRT-PCR in Calu-3 cells (Fig. S4). Serum albumin is a major component of lung lining fluid^47^. We hypothesize that the hypersecretion of serum albumin by Calu-3 cells a few days after ALI is related to the control of the osmotic pressure in an immature epithelium. This is supported by the secretion of Hsp90, a heat shock protein secreted by normal cells in case of tissue injury that also promotes cancer cell motility^48^. A basal level of secretion of serum albumin was then observed in both models.

At day 11 and 18 after ALI, similar features were observed in the apical secretome of Calu-3 cells, suggesting that the secretome becomes more stable after 7–10 days at ALI in this model. Proteins involved in the formation of the mucus layer such as MUCA5AC, and in cell communication and defense mechanisms, such as the neutrophil gelatinase associated lipocalin (*lcn2*), the polymeric immunoglobulin receptor (*pigr*), alpha-1-antitrypsin (*serpina1*) and alpha-1-antichymotrypsin (*serpina3*) became more abundant in the mature secretome of Calu-3 cells (Fig. 4a). Clusterin (*clu*), an extracellular chaperone protein associated with the folding of secreted proteins; galectin-3 binding protein (*lgals3bp*), a glycoprotein associated with the innate immune response of epithelial cells^49^; and annexin A2 (*anxa2*), which is involved in the cell secretory machinery, were also identified as main components of the mature secretome of Calu-3 cells. Annexin A2 can be trafficked across the membrane of the lung epithelial cells during the secretion of extracellular vesicles (EVs)^50^, which suggests that proteins identified in the apical secretome of Calu-3 cells include exosomal proteins^35^.

The comparison of the apical secretome of Calu-3 and NHBE cells shows that neutrophil gelatinase associated lipocalin, polymeric immunoglobulin receptor, clusterin, galectin-3 binding protein, and annexin 2 were secreted at high levels in both models. Other major components of NHBE cell secretome included uteroglobin (*scgb1a1*) and the BPI fold containing family A (*bpifa1*) and B (*bpifb1*), which are involved in the defense mechanisms of the epithelium (Fig. 4b).

### Comparison of the long-term evolution of Calu-3 and NHBE cell secretomes

**Figure 5:**
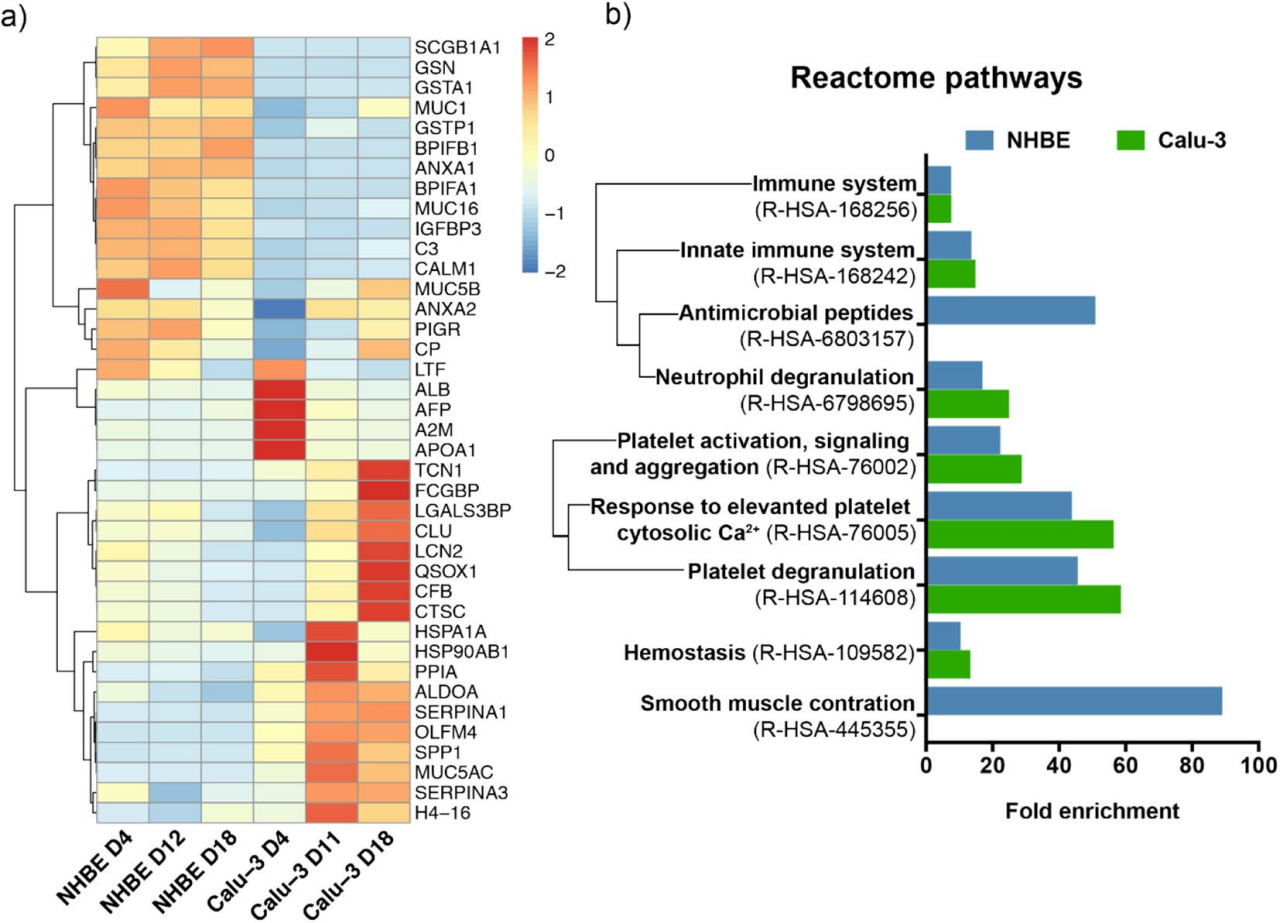
Analysis of the biological pathways associated with the secreted proteins in the Calu-3 and NHBE models. (**a**) Heat map of the 20 most abundant proteins secreted by Calu-3 and NHBE cells at day 4, 11–12, and 18 after ALI. Proteins are designated by their corresponding gene. (**b**) Reactome pathways associated with the extracellular proteins identified in the apical secretome of Calu-3 cells (in green) and NHBE cells (in blue) at day 11–12 and 18 after ALI. (P < 0.05 for each fold enrichment).

In order to better understand the long-term evolution of the epithelial cell secretome, the 20 most abundant proteins secreted by Calu-3 and NHBE cells (Top 20) were compared at each time point (Fig. 5a, Table S2). The heat map highlights the temporal evolution of each protein and the differences between the main components of the secretome of Calu-3 and NHBE cells.

The NHBE secretome was broadly stable for 3 weeks, except for a few proteins whose abundance tended to decrease a little (*muc5ab*, *muc1*, *serpina3*). Some proteins highly abundant in the NHBE secretome are secreted at lower levels by Calu-3 cells, such as gelsolin (*gsn*), glutathione S-transferases (*gsta*, *gstp1*), annexin 1 (*anxa1*), MUC16 (*muc16*), insulin-like growth factor-binding protein 3 (*igfbp3*), Complement C3 (*c3*) and calmodulin-1 (*calm1*). Gelsolin (*gsn*) breaks down actin filaments released by dying cells. Its secretion by epithelial cells is triggered by interleukin-4^51^. Gelsolin was detected at a lower level in Calu-3 secretome, suggesting a basal level of secretion of gelsolin in this model. Antimicrobial peptides were also secreted by NHBE cells, such as the BPI fold-containing family A and B members 1 (*bpifa1*, *bpifb1*).

The proteins secreted by Calu-3 cells can be clustered according to their time evolution during a period of 3 weeks: (1) highly secreted proteins during the first days at ALI, (2) highly secreted proteins in the mature secretome, (3) proteins with basal level of secretion. In addition, three proteins in the Top 20 showed a transient increase in secretion at day 11 after ALI: HSP70, HSP90, and the peptidyl prolyl isomerase A (*ppia*), which catalyses the folding of extracellular matrix proteins^52^.

The highly secreted proteins during the first days at ALI were serum albumin, α-2-macroglobulin (*a2m*), α-fetoprotein (*afp*), and apolipoprotein A1 (*apoa1*). The presence of α-fetoprotein was reported in the bronchoalveolar lavage (BAL) of patients with lung adenocarcinoma^53^, suggesting its secretion could be specific to the Calu-3 cell line in the immature epithelium. This hypothesis is consistent with the higher level of secretion of albumin at this time point. α-2-macroglobulin contributes to the control of protease activity in the lung lining fluid. The secretion of apoA1 by epithelial cells was recently linked to the regulation of the innate immune response following airway inflammation^54^.

Proteins more specific to the mature secretome of Calu-3 epithelial cells include the major gel-forming mucins MUC5AC and MUC5B, and to a smaller extent the tethered mucins MUC1 and MUC16^55^. MUC1 and MUC16 are anchored to the cell membrane, which can explain their lower abundance in the analysis of the secretome. Protease inhibitors [α1-antitrypsin (*serpina1*), α1- antichymotrypsin (*serpina3*)], proteases [cathepsin C (*ctsc*)], immunoglobulin G (*fcgbp*), Complement factor B (*cfb*), and olfactomedin4 (*olfm4*), a glycoprotein involved in the innate immune response, were also secreted at higher levels after 11 and 18 days at ALI in the Calu-3 model. To explore the biological role and interactions of the main components of the epithelial cell secretions further, we performed a Reactome pathway analysis of the Calu-3 and NHBE secretomes.

### Biological pathways associated with the secretome of Calu-3 and NHBE cells

The biological pathways associated with the secreted proteins were analysed using the Reactome database. We selected the proteins secreted both at day 11–12 and 18 after ALI for this analysis, which correspond to the proteins forming a stable and mature secretome in Calu-3 and NHBE models. The Reactome pathways associated with the secretome of Calu-3 and NHBE cells in long-term cultures are presented in Fig. 5b and Table 1. The secreted proteins are strongly associated with the immune response both in Calu-3 and NHBE models. This response involves the innate immune response, the neutrophil degranulation, and antimicrobial peptides (*bpifa1, bpifb1, clu, lcn2*)^56,57^. The proteins secreted by the epithelial cells, such as lipocalin 2, BPI fold containing family A and B members, and clusterin, are essential elements of the mucosal immune defense against pulmonary infections^58^.

**Table 1:** List of the biological pathways and their associated proteins identified in the secretome of NHBE and/or Calu-3 cells at day 11–12 and 18 after ALI.

Reactome pathway	Top 20 NHBE + Calu-3	Top 20 NHBE	Top 20 Calu-3
Immune system	*anxa2* *clu* *lcn2* *pigr* *serpina3*	*anxa1* *bpifa1* *bpifb1* *c3* *calm1* *gsn* *gstp1* *muc1* *muc16*	*cfb* *ctsc* *muc5ac* *olfm4* *serpina1* *tcn1*
Innate immune system	*anxa2* *clu* *lcn2* *pigr* *serpina3*	*bpifa1* *bpifb1* *c3* *calm1* *gsn* *gstp1* *muc1* *muc16*	*cfb* *ctsc* *muc5ac* *olfm4* *serpina1* *tcn1*
Antimicrobial peptide		*bpifa1* *bpifb1* *clu* *lcn2*	
Neutrophil degranulation	*anxa2* *lcn2* *pigr* *serpina3*	*c3* *gsn* *gstp1*	*ctsc* *olfm4* *serpina1* *tcn1*
Platelet activation, signalling and aggregation	*alb* *clu* *lgals3bp* *serpina3*	*calm1*	*serpina1*
Response to elevated platelet cytosolic Ca^2+^	*alb* *clu* *lgals3bp* *serpina3*	*calm1*	*serpina1*
Platelet degranulation	*alb* *clu* *lgals3bp* *serpina3*	*calm1*	*serpina1*
Homeostasis	*serpina3* *alb* *clu* *lgals3bp* *anxa2*	*calm1*	*serpina1*
Smooth muscle contraction		*anxa1* *calm1*	

Only the 20 most abundant proteins were considered. Proteins are designated by their corresponding gene. The description of each protein is given in Table S2.

The biological pathways associated with hemostasis and platelet activation include albumin and calmodulin-1 (*calm1*), a calcium-binding protein that modulates the ion channel activity. These proteins may contribute to the regulation of the osmotic pressure in the epithelium^59^.

### Expression of SARS-CoV-2 receptors in preclinical 3D model of the human bronchial epithelium

The infection of Calu-3 cells by SARS-CoV-2 was recently reported^60,61^. The ACE-2 (angiotensin-converting enzyme 2) receptor and the neuropilin-1 receptor, which are involved in SARS-CoV-2 infection of the lung epithelial cells^62,63^, were both expressed by Calu-3 cells for 4 weeks in our 3D model of human bronchial epithelium (Supplementary dataset 1). Proteomic analysis of cell extracts and qRT-PCR will be performed to confirm the levels of expression of these receptors in long-term Calu-3 cell cultures. The presence of these receptors could support the use of this preclinical model for testing novel therapeutic strategies against COVID-19^64–66^.

## Discussion

The objectives of this study were to develop a preclinical model of the human bronchial epithelium using the Calu-3 cell line for chronic toxicity study with minimal FBS supplementation, and to characterize the time evolution of the epithelial cell secretions. The Calu-3 cells were maintained for 21 days at ALI without subculturing and showed good viability and functionality, making it a relevant model for chronic toxicity studies of inhaled drugs, particles or pollutants. Here, we adapted the Calu-3 model for long-term nanotoxicology studies for repeated and chronic exposure to inhaled NPs by culturing cells on inserts with a 3-µm pore diameter. A larger pore size allows the analysis of NP translocation through the epithelial barrier^9^. Compared to primary cell models, the main advantages of the Calu-3 model are the availability, low cost, absence of donor variability, and the formation of a tight epithelium on inserts with a 3-µm pore diameter with excellent barrier properties without addition of any drug^15,21,22,67^. In comparison, the growth of NHBE cells is not possible on membranes with such a large porosity, and is limited to inserts with a 0.4-µm pore diameter. The disadvantages of the Calu-3 model are the presence of only one cell type, an abnormal karyotype, and the growth of polyps in certain conditions (data not shown). Some differences in anion secretion during transepithelial fluid transport were also reported^28^.

Another advantage of 3D models is the possibility to establish co-cultures to mimic cellular interactions between different cell types in vitro. For instance, co-cultures of the epithelial cells with macrophages and fibroblasts on the apical and basal side of the bronchial epithelium respectively were used to investigate the fibrogenic potential in toxicology studies^68^. Our long-term culture model could be of particular interest regarding the development of this pathology. Interestingly, in the case of silver NPs, Braakhuis et al. showed that a simple in vitro model could better predict the pulmonary toxicity when compared to in vivo inhalation studies than co-cultures with endothelial cells and macrophages^14^.

We determined a minimal FBS supplementation of 4% in the basal medium of Calu-3 cells to maintain a functional epithelium for 21 days at ALI. While cells cannot be deprived of serum, reducing the FBS supplementation is relevant in a lung model for testing drug or particle toxicity. On one hand, drugs may form complexes with serum proteins, reducing their bioavailability or triggering allergic responses; and NPs are covered by adsorbed proteins (the biomolecular corona) which defines their biological identity and cellular interactions. On the other hand, more than 1800 proteins and 4000 metabolites compose FBS, the concentrations of which vary greatly between batches^39^. Therefore, supplementation introduces a degree of complexity and variability that can have a substantial impact on the results. Here we advise to use minimal FBS supplementation for toxicity testing to reduce this risk. Future solutions may be provided with the use of serum substitutes and serum-free culture conditions.

On the apical side, the epithelial cell secretions structure the mucus that forms, with the tight epithelium, the first line of defense against particles and pathogens at the bronchial level^69^. The composition of the secretome and its evolution are therefore key elements of lung models. Our analysis of the apical secretome of Calu-3 and NHBE epithelia in long-term cultures shows high similarity between the two models, despite the difference in cell types. The same proteins are secreted, albeit with different abundances. While the NHBE secretome shows little variability with time, an evolution of Calu-3 secretions was clearly visible from 4 to 18 days at ALI. Based on this observation, we suggest that a stable and mature secretome is established after 11 days at ALI in the Calu-3 model.

The epithelial cell secretions, which include mucins and a large range of enzymes as well as antimicrobial peptides such as lipocalin 2, are key components of the mucosal immune defense against xenobiotics and pathogens. The Reactome biological pathway analysis shows their strong relation with the immune response of the epithelium. Proteins secreted by the Calu-3 and NHBE models of the bronchial epithelium were also identified in the human BAL of healthy individuals^70^. Shaw et al. showed that the biomolecular corona formed on diesel exhaust nanoparticles (DNEPs) in BAL induced an inflammatory response in macrophages and increased cell uptake, an effect unseen when the DNEPs were incubated in plasma^40^. Differences in the uptake of oxide NPs by rat alveolar macrophages in vivo was related by Konduru et al. to differences in their protein corona composition following incubation in rat lung lining fluid^41^. These studies highlight the role of the epithelial cell secretions on the mucosal immune response and on the fate of the inhaled particles.

Exosomal proteins, such as annexin A2, were also noted in the secretome of Calu-3 cells by Gupta et al., suggesting that extracellular vesicles EVs were produced by the bronchial epithelium in our model^50^. The proteomic analysis of the lung EVs was recently provided by Gupta et al. for the Calu-3 cell line^35^. The authors demonstrated that lung EVs played a central role in the epithelial cell communication in the upper airways. These results confirm the biological relevance of the Calu-3 model for long-term toxicity studies and the essential role of the epithelial cell secretions in the lung response.

Finally, diseases such as COPD and cystic fibrosis can alter mucus secretion^46^. The analysis of the BAL of atopic asthmatics showed altered proteomic profiles, which result in different biomolecular corona on inhaled particles^71^. The interaction of an inhaled agent (drug, particle, pollutant) with the mucus and its effect on mucus secretion are two essential aspects of the epithelial response, especially for chronic exposure. The secretome profile provided here can be used as a reference to investigate the effect of drugs on the epithelial cell secretions over time.

Taking advantage of microfabrication techniques, advanced lung models such as lung-on-chip have been developed to recapitulate other biological lung features such as breathing cycle, deep lung morphology, and airflow in the alveoli^72,73^. Using the Calu-3 cell line, functional and mucus producing lung-on-chip models could be developed to investigate the effect of exogenous compounds on mucus evolution and/or alteration in vitro. The immortalized BSi-NC1 cells developed by Crystal’s group also represent an interesting model to establish a tight epithelium with both ciliated and non-ciliated cells following differentiation at ALI^74^.

To conclude, a functional in vitro model of the human bronchial epithelium using Calu-3 cell line was developed as an alternative to primary NHBE cells. A minimal FBS supplementation in the basal medium was defined to maintain a functional epithelium for 21 days at the air liquid interface, so that the amount of exogenous serum proteins could be reduced during drug testing. The nature and the biological pathways associated with the secreted proteins confirmed their key role in the mucosal immune response of the lung. We suggest this preclinical 3D model can be used to evaluate the long-term toxicity of drugs or particles on the human bronchial epithelium, and subsequently to investigate their effect on the epithelial cell secretions.

## Methods

### 3D model of the human bronchial epithelium with Calu-3 cell line

The Calu-3 human adenocarcinoma epithelial cell line (ATCC HTB-55, LGC Standard, France) was used for the experiment at cell passage 24–40. All products used for cell culture were provided by Thermo Fisher Scientific unless stated otherwise. Calu-3 cells were cultured in Eagle’s Minimum Essential Medium (MEM) supplemented with 10% v/v fetal bovine serum (FBS) (F7524, Sigma-Aldrich), 1% non-essential amino acids (NEAA) 100×, 1% sodium pyruvate, 1% Glutamax, 1% penicillin streptomycin 100×, and 1% HEPES buffer 100×. Cells were culture at a density of 40,000 cells/cm^2^ in 25 or 75 cm^2^ culture flasks (Corning) at 37 °C in a humidified 5% CO_2_ atmosphere and passaged weekly before confluence. To form the epithelial barrier, Calu-3 cells were seeded on Transwell polyester inserts with a 3 µm pore diameter (Corning Costar) at a density of 500,000 cells/insert (with 500 µL of cell suspension in the apical compartment and 1.5 mL of medium in the basolateral compartment). The culture medium was changed every 2–3 days in both compartments until TEER > 700 Ω cm^2^. The medium was removed from the apical compartment to create an air–liquid interface. The medium in the basolateral compartment was replaced by 0, 2, 4 or 8% FBS one day after ALI. Calu-3 cells cultured on Transwell membrane were maintained for 21 days after ALI.

### MucilAir 3D lung tissue model

MucilAir 3D lung tissue model is a fully differentiated bronchial epithelium reconstituted from human bronchial airway epithelial cells of healthy donors. The MucilAir cultures were purchased from Epithelix (Genève, Switzerland). Cells were obtained from 3 healthy non-smoking Caucasian donors, two men and one woman, of different ages. The primary cells were cultured on Transwell polyester inserts with 0.4 µm pore diameter using MucilAir serum-free cell culture medium with penicillin/streptomycin (EP04MM, Epithelix).

### Trans-epithelial electrical resistance

To assess the integrity of the epithelial barrier, the trans-epithelial electrical resistance (TEER) was measured with an EVOM2 ohmmeter (World Precision Instruments, Sarasota, USA) with STX2 electrodes. First, the culture medium was replaced by 1.5 mL and 0.5 mL Hanks’ Balanced Salt Solutions supplemented with calcium and magnesium (HBSS^Ca2+/Mg2+^, ThermoFisher Scientific, France) in the basal and apical compartment, respectively. HBSS was warmed at 37 °C before addition. For each experiment, the TEER was also measured in a cell-free Transwell (blank) and subtracted to values measured in cell seeded Transwell. Final TEER values are multiplied by the surface area of the inserts and expressed in Ω cm^2^. We considered that a tight epithelium was formed if TEER > 200 Ω cm^2^ for MucilAir following the provider’s guidelines, and > 300 Ω cm^2^ for Calu-3 cells. This latter value was determined by combining TEER and Lucifer Yellow permeability assay results in our cell culture conditions (Fig. S2).

### Lucifer Yellow permeability assay

The integrity of the barrier formed by Calu-3 cells at the air–liquid interface was checked with the Lucifer Yellow (LY) paracellular permeability assay (Sigma-Aldrich). LY is a 452 Da fluorescence dye which paracellular transport through the epithelium is prevented when tight junctions are formed (in the case of a short exposure time of the cell layer to LY). 0.5 mL LY (0.1 mg/mL), diluted in HBSS^Ca2+/Mg2+^, were added to the apical compartment. The basal compartment was filled with 1 mL HBSS^Ca2+/Mg2+^. LY was also added to a cell free well (blank). Following 1 h incubation at 37 °C, 0.1 mL of the apical and basal solutions were transferred to a 96-well microplate (µClear, Greiner Bio-One, Germany). The fluorescence intensity was measured with λ_exc_ = 485 nm and λ_em_ = 535 nm on a Flex microplate reader (Molecular Devices). This analysis was performed in triplicate. The permeability was calculated as follow:

$$Permeability\;\left( \% \right) = \frac{{I_{sample} - I_{Blank} }}{{I_{LY} - I_{Blank} }} \times 100$$

where I_LY_, I_sample_, and I_blank_ are the fluorescence intensities of the dye, the sample and the blank (cell-free sample) respectively. A permeability of < 2% was used as a threshold to define a tight epithelium.

### BCA assay

The total protein concentration of the apical secretome was measured by the bicinchoninic acid (BCA) protein assay (Pierce BCA protein assay kit, ThermoFisher Scientific) following the manufacturer’s protocol. Briefly, all samples were diluted in HBSS^Ca2+/Mg2+^. After 30 min incubation at 37 °C, the absorbance at 562 nm was measured on the Flex microplate reader. The analysis was performed in duplicate.

### Enzyme-linked lectin assay

The glycoprotein concentration of the apical secretome of Calu-3 cells and MucilAir was measured by the enzyme-linked lectin assay (ELLA). A 96-well plate (9018, Corning) was coated with 6 µg/mL lectin from *Triticum vulgaris* (L0636, Sigma-Aldrich) in PBS (ET330-A, Euromedex) and incubated for 1 h incubation at 37 °C. After washing the plates with PBS buffer supplemented with 0.5 M NaCl (S3014, Sigma-Aldrich) and 0.1% Tween 20 (P1379, Sigma-Aldrich), 50 µL of the apical secretome were added and incubated for 1 h at 37 °C. Known concentration of porcine stomach mucin (2378, Sigma-Aldrich) were added to the same plate to measure the calibration curve [78.1 ng/mL–10 µg/mL]. After washing, the detection solution composed of 1 µg/mL lectin peroxidase conjugated (L2650 Sigma-Aldrich) was added to each well and incubated for 1 h at 37 °C. Finally, tetramethylbenzidine (TMB) substrate reagent (555214 BD OptEIA, BD Bioscience) was added and incubated for 20 min in the dark at room temperature. The enzymatic reaction was stopped with 2 N H_2_SO_4_ (84727, Sigma-Aldrich) and the absorbance at 490 nm was measured. Each analysis was performed in duplicates.

### MUC5AC and ZO-1 immunolabelling

Cells were fixed with 4% paraformaldehyde, washed with PBS, permeabilized with a 0.02% Triton X-100 (2000B, Euromedex), and blocked with 2% BSA (A7906, Sigma-Aldrich) in PBS. After washing, cells were incubated with mouse monoclonal anti-MUC5AC (dilution 1/500) (45M1-12178, Thermo Fisher scientific) and rabbit polyclonal anti-ZO-1 (SC-10804, Santa Cruz Biotechnology) antibodies at 4 °C overnight. Donkey anti-mouse-AF594 (715-585-150) and goat anti-rabbit-AF488 (111-545-144) secondary antibodies (Jackson ImmunoResearch) were used (dilution 1/1000). Cells were mounted with Fluoroshield (F6057, Sigma-Aldrich) before observation by confocal ZEISS LSM 700 fluorescence microscope with a 63× objective lens. Version 2.3 of the ZEISS ZEN software was used.

### Transmission electron microscopy

Calu-3 cells cultured on Transwell were fixed with 2.5% glutaraldehyde and 2% paraformaldehyde in 0.1 M sodium cacodylate pH 7.3 for 45 min at room temperature. The samples were then treated with 1% osmium tetroxide for 45 min at 4 °C, and incubated in 1% aqueous uranyl acetate solution for 2 h at room temperature. They were dehydrated in ethanol solutions of increasing percentage (30%, 50%, 70%, 95% and 100%, 3 × 10 min each), in ethanol:propylene oxide mix (1:1 v), and in propylene oxide 3 × 10 min. Each sample was embedded in Epon epoxy resin. Ultrathin sections of 80 nm thickness were cut with a Leica Ultracut S microtome fitted with a diamond knife (Diatome ultra 45), transferred on Cu grids and post-stained with lead citrate. Samples were imaged with a JEM-100S microscope (Jeol Ltd Tokyo, Japan) operating at 80 kV. Images were acquired with an Orius 200 digital camera (Gatan-Roper Scientific, Evry, France) using Gatan software.

### qRT-PCR

RNA was isolated from Calu-3 cells 20 days after ALI using NucleoSpin RNA kit (740955.250, Macherey–Nagel) and converted to cDNA using the high-capacity cDNA reverse transcription kits (4368814, Applied Biosystems). Primers for the selected genes of interest (*muc5ac, muc5b, zo1, alb*) were designed with Primer-Blast software (NCBI) and synthesized by Eurofins Genomics (*zo-1, hprt, muc5ac*), Oligo (*tbp*) and Invitrogen (*muc5b*). The sequence of the primers is detailed in Table S1. qRT-PCR was performed on a LightCycler 480 instrument II (Roche Diagnostics, France). Gene expression was analyzed by 2^−ΔΔCt^ method and normalized to RP19 and TBP housekeeping genes. Fold change was expressed using 10% FBS cell culture condition as the reference. Results are expressed as mean fold change ± standard deviation for three biological replicates.

### LC–MS/MS

Label-free quantitative proteomic analysis of the Calu-3 and NHBE apical secretome was performed by LC–MS/MS following protein digestion with trypsin. 3 biological replicates were analyzed at day 4 and day 11, 2 biological replicates were analyzed at day 18. One biological replicate corresponds to the secretome collected from three different Transwell from the same donor pulled together in the case of the NHBE model, and to the secretome collected from three different Transwell from two different culture batches in the case of the Calu-3 model. Briefly, 16 µg of protein were precipitated in cold acetone, then resuspended in 25 mM NH_4_HCO_3_ buffer prior to tryptic digestion overnight (sequencing-grade Trypsin, Promega). Peptides were desalted and concentrated with 10 μL ZipTip µ-C18 Pipette Tips (Millipore,). Peptides were analyzed on a Q-Exactive Plus mass spectrometer coupled to a Proxeon 1000 Nano-LC (ThermoFisher). Peptides were separated by chromatography using the following specifications: acclaim PepMap100 C18 pre-column (2 cm, 75 μm i.d., 3 μm, 100 Å); Pepmap-RSLC Proxeon C18 column (50 cm, 75 μm i.d., 2 μm, 100 Å); 300 nL/min flow rate, 98 min gradient going from 95% solvent A (water, 0.1% formic acid) to 35% solvent B (100% acetonitrile, 0.1% formic acid) followed by column regeneration (total time 120 min). Peptides were first analyzed in positive mode in the Orbitrap cell at a 70,000 resolution with a m/z range of 375–1500. MS/MS data were acquired in the Orbitrap cell in a Top20 mode with an AGC target of 3.10^6^ for full MS. Fragments were obtained by Higher-energy C-trap Dissociation (HCD) activation with a collisional energy of 27%, a quadrupole isolation window of 1.4 Da, and an AGC target of 2.10^5^. MS/MS data were acquired in a data-dependent mode with a dynamic exclusion of 30 s. Monovalent peptides or peptides with unassigned charge state were excluded from the analysis. The maximum ion accumulation times were set to 50 ms and 45 ms for MS and MS/MS acquisition respectively. Label Free quantitation was performed with Progenesis QI (Waters) using HI-3 method for protein quantification. Data were processed with Proteome Discoverer 2.2 software (ThermoFisher Scientific). The mass tolerance was set to 6 ppm for precursor ions and 0.02 Da for fragments.

### Proteomic data analysis

The MASCOT software (Matrix Science, v. 2.4) was used for protein identification on the *Homo sapiens* and *Bos Taurus* Swissprot databases (2019). Post-translational modifications were searched in dynamics parameters: oxidation (M) phosphorylation (S/T/Y), acetylation (Protein N-terminal). The maximum number of missed cleavages was limited to two for trypsin digestion. P-values of peptides were calculated using the percolator algorithm and a 5% threshold was applied. Filters used in the MASCOT software correspond to: proteins identified with a minimum of 2 peptides, AND a MASCOT score > 40, AND P < 0.05. Extracellular proteins were identified using the extracellular compartment database of the Proteome Discoverer software. Bioinformatics analysis and figures were developed with R Software (v.3.6.2)^75^. Heat maps were generated with the ‘pheatmap’ package^76^, where correlation clustering distance row was applied. PCA was calculated from the abundance of the extracellular proteins identified in each replicate and the results presented using the ‘ggbiplot’ package^77^. The ‘packcircles’ package^78^ was used to represent protein abundance, where the circle area is proportional to each protein abundance. The Reactome pathway fold enrichment analysis was performed with PANTHER (v15.0) software using Fisher’s test and Bonferroni correction for multiple testing. Statistically significant results were selected using P < 0.05.

### Statistical analysis

The statistical analysis was performed with Prism GraphPad Software (v. 7.0). Data are expressed as mean ± standard deviation. All data passed the D’Agostino and Pearson or the Shapiro–Wilk normality test (α = 0.05). When comparing groups, multiple comparison two-way ANOVA corrected with Benjamini, Krieger and Yekutieli test were used. For comparing time variability within group, three-way ANOVA corrected with Benjamini, Krieger and Yekutieli test were used. For Lucifer yellow assay, Boneferroni-Dunn t-test method was applied. P < 0.05 was used as a threshold for statistically significant results.

## Supplementary Information

Supplementary Information.

## Data Availability

The mass spectrometry proteomics data have been deposited to the ProteomeXchange Consortium via the PRIDE partner repository^79^ with the dataset identifier PXD024242. The full protein lists are also available in the Excel file WS1 in the “SI”.
