# Supplementary material for: Long-term evolution of the epithelial cell secretome in preclinical 3D models of the human bronchial epithelium
Source: Sci Rep. 2021 Mar 23;11:6621. doi: 10.1038/s41598-021-86037-0 (PMC7988136; doi:10.1038/s41598-021-86037-0)
Supplement: Supplementary file 1 — Supplementary Information. [file 41598_2021_86037_MOESM1_ESM.pdf]

Supplementary Information

Long-term evolution of the epithelial cell secretome in preclinical 3D models of the human bronchial epithelium

Daniel Sanchez-Guzman, Sonja Boland, Oliver Brookes, Claire Mc Cord, René Lai Kuen, Valentina Sirri, Armelle Baeza Squiban, Stéphanie Devineau*

Table of Contents

1. Variability of the trans-epithelial electrical resistance in Calu-3 cultures
2. Comparison of TEER measurement and Lucifer Yellow permeability assay
3. EdU cell proliferation assay
4. Gene expression in Calu-3 cells 20 days after air-liquid interface (Table S1)
5. TEM images of Calu-3 cells at the air-liquid interface
6. Comparison of the 20 most abundant proteins in the secretome of Calu-3 and NHBE cells at day 4, day 11, and day 18 (Table S2)

Associated files

The full list of proteins identified in the apical secretome of Calu-3 and NHBE cells at day 4, 11-12, 18 after ALI is provided in Excel file WS1.

1. Variability of the trans-epithelial electrical resistance in Calu-3 cultures

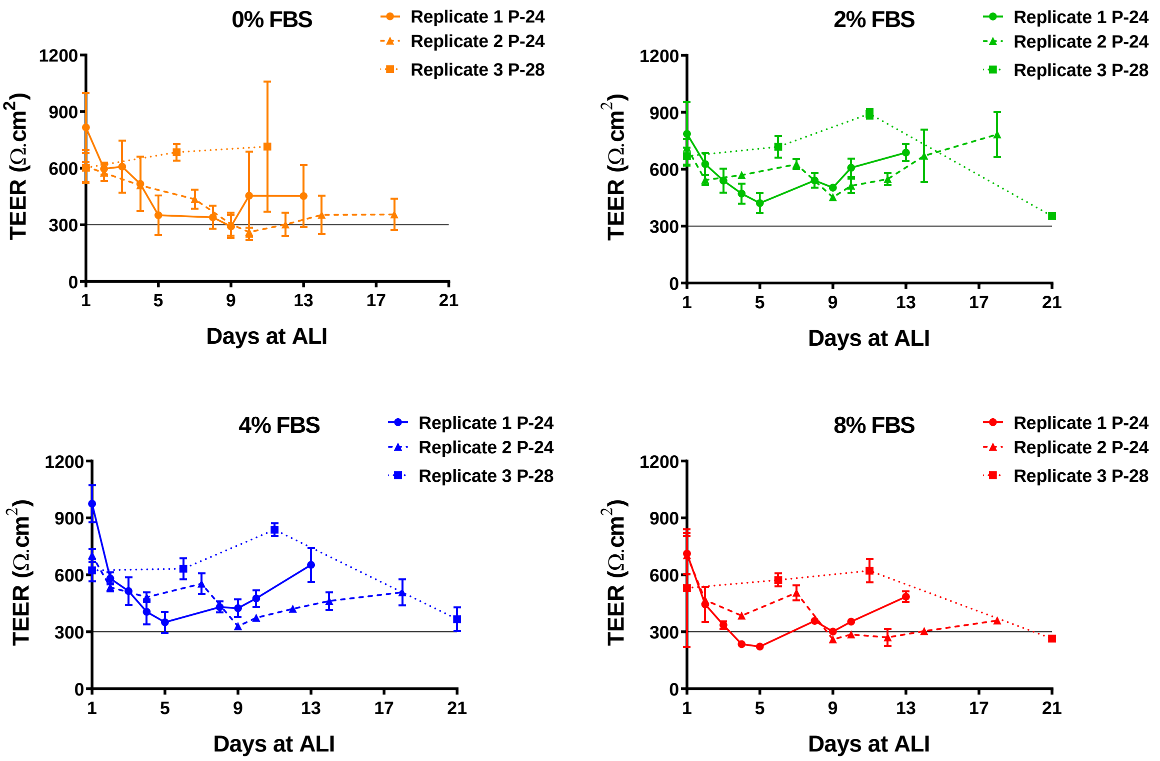

Figure S1. Comparison of the trans-epithelial electrical resistance measurement for 3 biological replicates with 0% (a), 2% (b), 4% (c), and 8% (d) FBS in the basolateral medium. Cells were kept at ALI for 21 days. The number of passages of Calu-3 cells is indicated for each experiment. Replicates 1 and 2 were carried out with Calu-3 cells at passage 24 (P-24). Replicate 3 was carried out with Calu-3 cells at passage 28 (P-28). The black line indicates the minimum TEER value for a tight epithelium used in this study (300 Ω.cm²). Results are presented as mean ± standard deviation (n = 3).

2. Comparison of TEER measurement and Lucifer Yellow permeability assay

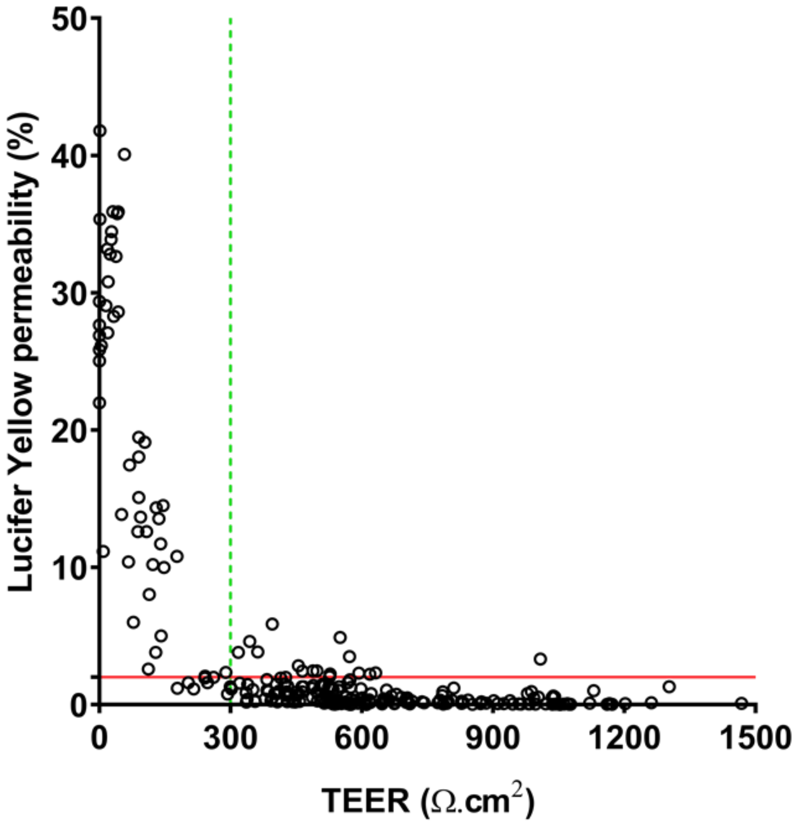

Figure S2. Comparison of TEER measurement and Lucifer yellow permeability assay for Calu-3 cells at ALI. The TEER and LY permeability were measured on 236 different samples with tight or leaky epithelial barrier. The red line corresponds to the maximum LY permeability defined by the manufacturer for a tight epithelium (LY < 2%). The green dotted line corresponds to the minimum TEER value we defined for a tight epithelium in our experimental conditions based on these data (TEER > 300 Ω.cm²).

3. EdU cell proliferation assay

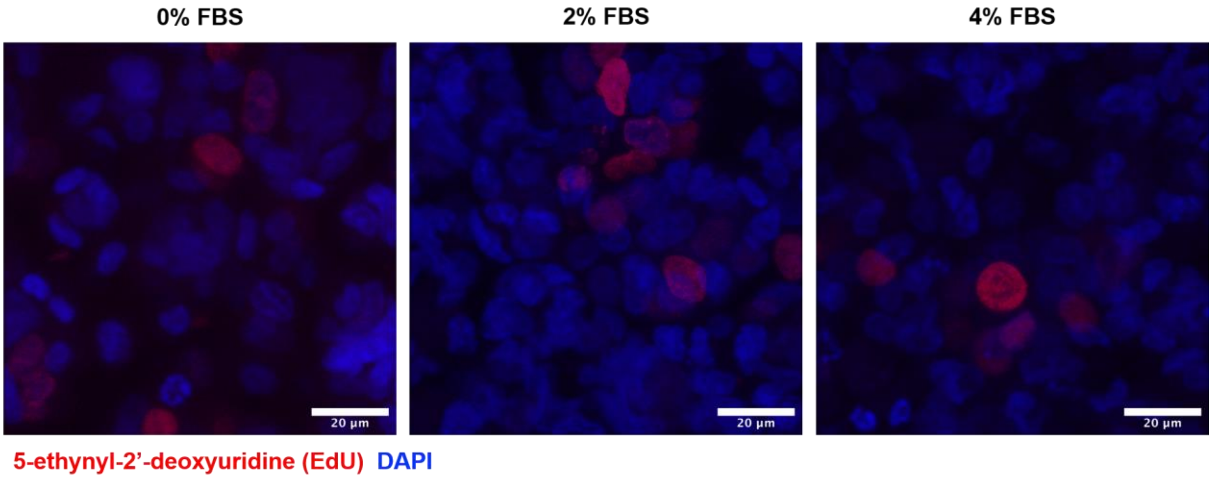

Figure S3. EdU cell proliferation assay performed on Calu-3 cells at ALI with 0, 2, 4% FBS supplementation in the basal medium. Fluorescence microscopy images with EdU positive cells in red and DAPI staining in blue. Scale bar is 20 µm.

4. Gene expression in Calu-3 cells 20 days after ALI

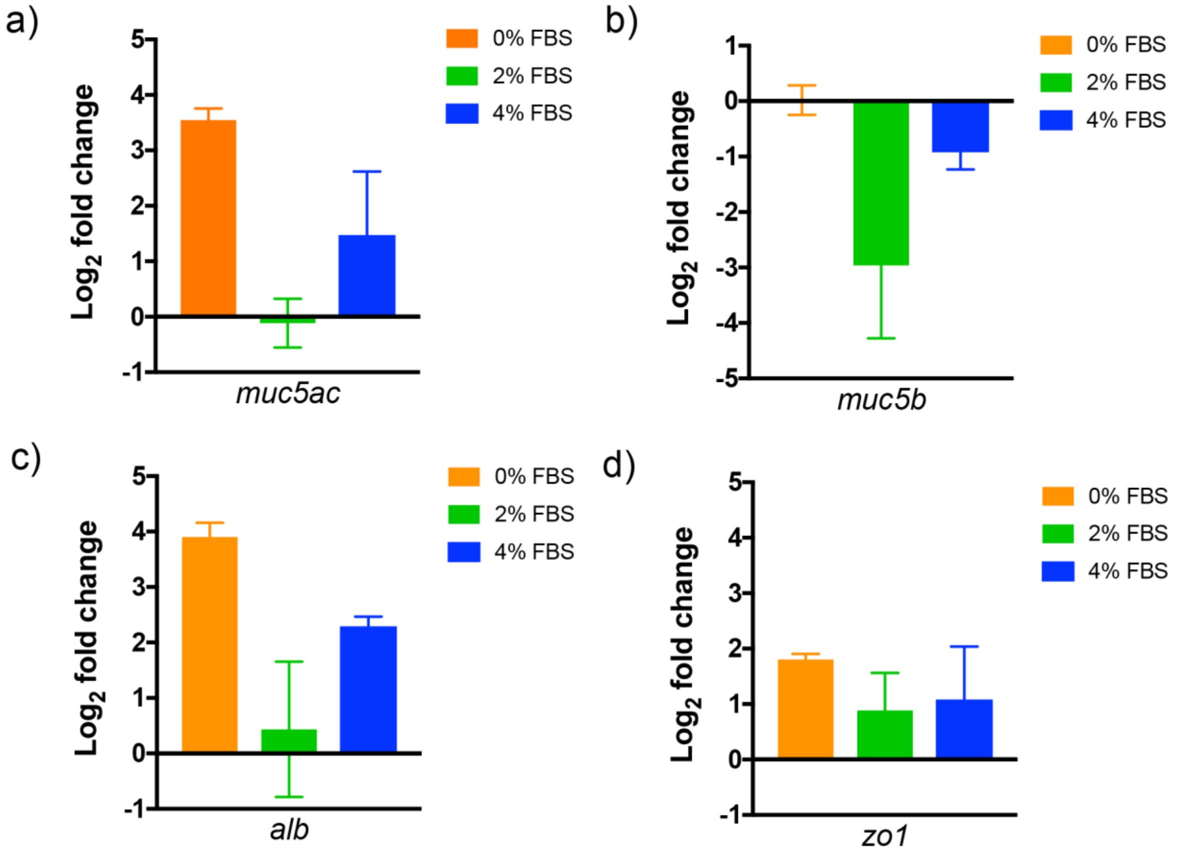

Figure S4. Gene expression measured in Calu-3 cells 20 days after ALI by qRT-PCR for *MUC5AC*, *MUC5B*, *ZO-1* and *ALB* as a function of the FBS percentage in the basolateral medium 0% (blue), 2% (brown), 4% (green). Gene expression was analyzed by $2^{-\Delta\Delta Ct}$ method and normalized to RP19 and TBP housekeeping genes. Fold change was expressed using 10 % FBS cell culture as the reference. Results are expressed as mean ± standard deviation for 3 biological replicates.

Table S1. Sequence of the primers used for qRT-PCR

	Forward	Reversed
alb	TAGAGAAGTGCTGTGCCGCT	AGTTGGAGTTGACACTTGGGGT
muc5ac	AACTACTCCCAGCCCTGTTC	GTAGAGGCAGGGGTTGTTCT
muc5b	GGGATCTTCCTGGTCATCGA	GCTACGCGTGGCAAAGTCAT
zo1	TGCAGCCAAGGAAGGCTTAGA	GGTCAAGCAGGAAAAGGACGG
tbp	GTGATCTTTGCAGTGACCCAG	ATAGACAGACTATTGGTGTTCT
rp19	GGCTCGCCTCTAGTGTCCTC	CAAGGTGTTTTTCCGGCATC

5. TEM images of Calu-3 cells at the air-liquid interface

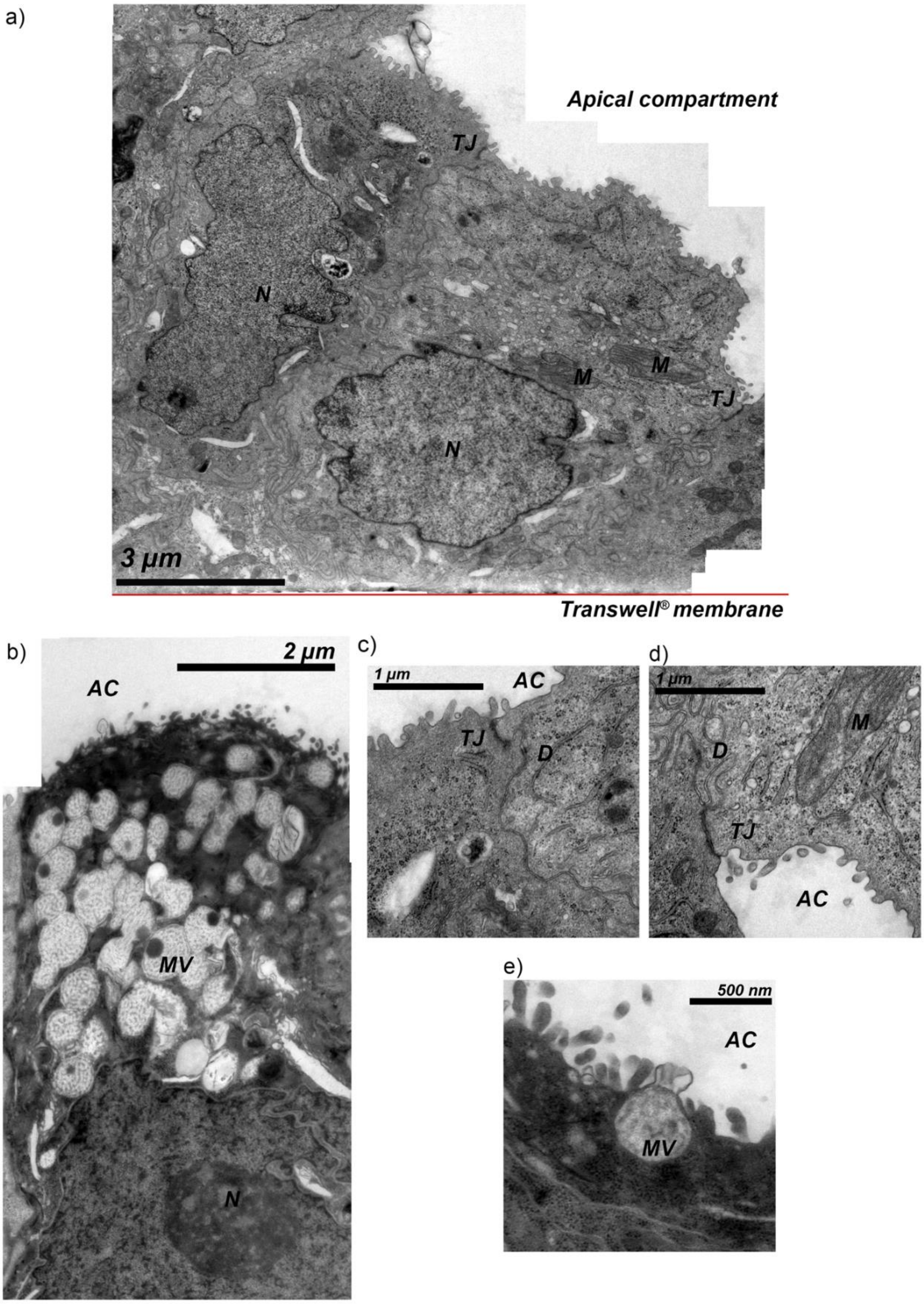

Figure S5. Transmission electron microscopy images of Calu-3 cells at the air-liquid interface. Cells were cultures with minimal FBS supplementation (4% in the basal medium) and fixed 8 days after ALI. (a) Overview of the bronchial epithelium with the Transwell membrane at the bottom and the apical surface at the top right. (b) Mucus vesicles located in the apical part of the epithelium. (c-d) Tight junctions and desmosomes. (e) Secretion of a mucus vesicle at the apical surface. N nucleus, TJ tight junction, D desmosome, M mitochondria, MV mucus vesicles, AC apical compartment.

6. Comparison of the 20 most abundant proteins in the secretome of Calu-3 and NHBE cells at day 4, day 11, and day 18

Table S2. List of the 20 most abundant proteins (Top 20) in the secretome of Calu-3 and NHBE cells at day 4, 11-12, and 18 after ALI. + indicates the protein was identified in the Top 20 in this condition (in blue for NHBE secretome, in green for Calu-3 secretome).

Accession number	Description	Gene	NHBE			Calu-3		
			4	12	18	4	11	18
P01024	Complement C3	*c3*	+	+	+			+
P01009	Alpha-1-antitrypsin	*serpina1*				+	+	+
P01011	Alpha-1-antichymotrypsin	*serpina3*	+	+	+	+	+	+
P98088	Mucin5AC	*muc5ac*				+	+	+
P08238	Heat shock protein 90	*hsp90ab1*					+	
P01833	Polymeric immunoglobulin receptor	*pigr*	+	+	+	+	+	+
Q8TDL5	BPI fold-containing family B member 1	*bpifb1*	+	+	+			
P0DMV8	Heat shock protein 70	*hspa1a, hspa1b*					+	
P07355	Annexin A2	*anxa2*	+	+	+	+	+	+
P10451	Osteopontin	*spp1*				+	+	+
Q8WXI7	Mucin16	*muc16*	+	+	+			
P00751	Complement factor B	*cfb*	+	+		+	+	+
P10909	Clusterin	*clu*	+	+	+	+	+	+
P06396	Gelsolin	*gsn*	+	+	+			
Q08380	Galectin-3 binding protein	*lgals3bp*	+	+	+	+	+	+
Q6UX06	Olfactomedin-4	*olfm4*				+	+	+
P80188	Neutrophil gelatinase associated lipocalin	*lcn2*	+	+	+	+	+	+
P04075	Fructose bisphosphate aldolase A	*aldoa*				+	+	
P02768	Serum albumin (Homo sapiens)	*alb*	+	+	+	+	+	+
P02788	Lactotransferrin	*ltf*	+			+		
P09211	Glutathione S-transferase P	*gstp1*	+	+	+		+	
P04083	Annexin A1	*anxa1*		+	+			

P62937	Peptidyl prolyl isomerase A	*ppia*				+	+	
P20061	Transcobalamin1	*tcn1*				+	+	+
P15941	Mucin1	*muc1*	+	+	+			+
P62805	Histone H4	*h4-16*					+	
P0DP23	Calmodulin1	*calm1*	+	+	+			
Q9NP55	BPI fold-containing family A member 1	*bpifa1*	+	+	+			
P17936	Insulin-like growth factor-binding protein-3	*igfbp3*	+	+	+			
P02647	Apolipoprotein A1	*apoa1*				+		
P01023	Alpha-2 macroglobulin	*a2m*				+		
P08263	Glutathione S-transferase A1	*gsta1*			+			
P11684	Uteroglobin	*scgb1a1*	+	+	+			
P02771	Alpha-fetoprotein	*afp*				+		
Q9HC84	Mucin-5B	*muc5b*	+					+
Q9Y6R7	IgG Fc-binding protein	*fcgbp*						+
P00450	Ceruloplasmin	*cp*						+
O00391	Sulfhydryl oxidase 1	*qsox1*						+
P53634	Dipeptidyl peptidase 1	*ctsc*						+
P36955	Pigment epithelium-derived factor	*serpinf1*				+		
P62328	Thymosin beta-4	*tmsb4x*				+		
